# Defining periodontal health

**DOI:** 10.1186/1472-6831-15-S1-S6

**Published:** 2015-09-15

**Authors:** Angelo Mariotti, Arthur F Hefti

**Affiliations:** 1 Division of Periodontology, College of Dentistry, The Ohio State University, Columbus, Ohio, 43210, USA

## Abstract

Assessment of the periodontium has relied exclusively on a variety of physical measurements (e.g., attachment level, probing depth, bone loss, mobility, recession, degree of inflammation, etc.) in relation to various case definitions of periodontal disease. Periodontal health was often an afterthought and was simply defined as the absence of the signs and symptoms of a periodontal disease. Accordingly, these strict and sometimes disparate definitions of periodontal disease have resulted in an idealistic requirement of a pristine periodontium for periodontal health, which makes us all diseased in one way or another. Furthermore, the consequence of not having a realistic definition of health has resulted in potentially questionable **recommendations**. The aim of this manuscript was to assess the biological, environmental, sociological, economic, educational and psychological relationships that are germane to constructing a paradigm that defines periodontal health using a modified wellness model. The paradigm includes four cardinal characteristics, i.e., 1) a functional dentition, 2) the painless function of a dentition, 3) the stability of the periodontal attachment apparatus, and 4) the psychological and social well-being of the individual. Finally, strategies and policies that advocate periodontal health were appraised.

I'm not sick but I'm not well,

and it's a sin to live so well.

Flagpole Sitta, Harvey Danger

## Introduction

Most people use the word “health” casually, in juxtaposition to disease, with no frame of reference. Frequently used terms associated with health include “health benefits”, “health promotion”, “health prevention”, “health care”, “health insurance”, and “oral health”, to name just a few. Clearly, the word health means different things to different people in different situations. An epidemiologist may use mortality data to study the “health” of a population, the economist may discuss the “health” of the economy as it relates to sustainable GDP growth, the stressed-out dental student may wonder whether the final examinations will affect her “health”, most likely meaning her sanity, and periodontists are measuring attachment and bone levels when seeking information about periodontal “health". Obviously, these professionals use the same word but with very different meanings. Etymologically, the word “health” was derived from the Old English “hale”, meaning wholesome, sound, or well. By and large, the original and broad connotation of this word has prevailed but with modern context.

Although the original Old English meaning of health has survived through the centuries, what constitutes health in the twenty-first century is far more perplexing than we can suppose. What we consider to be healthy has evolved as a result of definitions of health that have varied with the times because society's perception of disease and health have been influenced by our expanding scientific knowledge base as well as our cultural, social, and individual value judgments [1,2]. Despite the continually changing set of pretexts, the definition of health is important because it provides a common reference point to define recurring signs and symptoms that are within a significant standard definition of normal. Therefore, instead of trying to define a set of periodontal diseases whose etiology we cannot comfortably explain, perhaps, a better policy would be to characterize what periodontal health actually constitutes and what can affect it. The goal of the present paper is to present a framework for periodontal health that may be useful in clinical decision-making.

## Definitions of health and the wellness - illness continuum

Any medical action, including dental, is ultimately in pursuit of some aspect of health. So, how does one define health? In the Preamble of the Constitution of the World Health Organization [3,4], health was described as “a state of complete physical, mental and social well-being and not merely the absence of disease or infirmity.” Furthermore, the Preamble postulates “enjoyment of the highest attainable standard of health is one of the fundamental rights of every human being.” Hence, for the WHO, complete health is the ultimate visionary endpoint that people and society should strive to achieve. At the Ottawa Charter for Health Promotion (1986) the WHO added that health is a resource for everyday life, not the objective of living. In summary, health was described as a positive concept emphasizing social and personal resources as well as physical capacities.

It is immediately apparent that in everyday life, the WHO definition is neither useful nor operationally helpful, since it is almost impossible to achieve. Furthermore, questions regarding the meaning of “complete physical and mental health” and “absence of disease or infirmity” have not been and probably never will be answered satisfactorily. As a result, scholars have offered their conceptual interpretations of health, which can be summarized in two broad categories: 1) the natural concept of health and 2) the holistic concept of health.

The natural concept of health [5] postulates that a person is healthy only if all organs function within defined limits, given a statistically normal environment. Disease is defined as the subnormal functioning of one or more organs. In this model, health and disease are mutually exclusive; in other words, a person is healthy when he or she is not diseased. Determining whether a person is healthy or diseased is decided primarily on information accrued from actuarial tables and/or clinical studies.

The holistic concept of health [6] claims that a person is healthy if he or she had the ability, given standard circumstances, to reach all of his or her vital goals. Standard circumstances should not be confused with normal circumstances, rather they relate to a cultural norm. Therefore, a person who has experienced high blood pressure for a long time yet continues to accomplish all of her desired day-to-day activities would be considered healthy. Placed into a dental context for illustration only, a person with stable gingival recession who can chew effectively without pain, and is not expressing an esthetic concern, would be considered periodontally healthy.

In periodontology, many disease classifications have been presented over the years. They share a common application of the natural health concept and define periodontal health as absence of any clinical signs of current or past disease. The American Academy of Periodontology (AAP) has defined health as “The condition of a patient when there is function without evidence of disease or abnormality” (AAP 2001). Applied exclusively to a periodontal framework, this definition prescribes absence of signs and symptoms of gingival and destructive periodontal diseases, or any tissue status outside the normal range. As a result, this idealistic requirement of a pristine periodontium makes us all diseased in one way or another.

One must ask if it is realistic to believe that periodontal health is ever achievable, or are there more sensible and evidence-based ways to view periodontal health? Considering the pretext for the natural concept of periodontal health as quixotic, could a view considered similar to health but different in scope, such as the concept of wellness, be beneficial? Wellness is considered a dynamic process involving an individual's positive and enthusiastic attitudes and behaviors that improve and ultimately maximize the quality of their life. Managing these feelings requires a realistic appraisal of coping mechanisms, autonomy concerns as well as understanding one's limitations. For example, a paraplegic can experience a state of wellness without having the ability to physically walk. Similar to wellness, illness is a highly personal state of being; however, unlike wellness, it is a condition in which the person feels unhealthy or ailing, and these feelings may or may not be related to a disease. Therefore, wellness and illness should be considered anchor points on a continuum reflecting a balance (or an imbalance) of mind, body and spirit.

**Figure 1:**
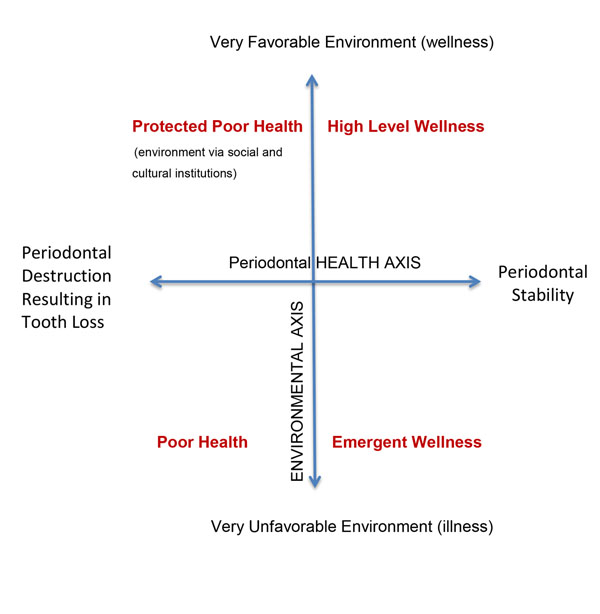
Periodontal Wellness Grid (Adapted from Dunn HL[161]).

In regard to the periodontium, wellness would be a dynamic state that varies from day-to-day. Each person would have an individual interpretation of their level of periodontal wellness (i.e., health), depending on personal values and cultural orientations. Such a definition of periodontal wellness is clearly different from the traditional biomedical approach (i.e., natural concept), which defines periodontal health as a complete absence of signs of disease. Using our current knowledge base as well as cultural standards and values, the proposed definition of a healthy periodontium, in a state of wellness, should include simple characteristics that are coherent in allowing an individual to attain positive goals consistent with a positive quality of life (Figure 1). These simple characteristics of periodontal wellness would include four cardinal components. They are the foundation for a proposed periodontal health model that includes aspects affecting the wellness-illness continuum.

## Assessing periodontal health

The most recent classifications of periodontal diseases published by the AAP and case definitions [7-9] were based on measurements of attachment level, probing depth, bone loss and/or degree of inflammation. Health (or “no disease") was defined as concurrent absence of these signs and symptoms. Accordingly, applying these strict and sometimes disparate definitions of periodontal disease has resulted in an unrealistically high estimated prevalence of periodontal disease throughout the world [10]. The consequence has been that a large portion of the adult population was identified with signs and symptoms of this disease. Most of these people function normally periodontally and do not feel ill. Still, often irrelevant and not sufficiently pragmatic health policy guidelines were established as a result of these definitions. It needs to be recognized that periodontal health, as defined by the AAP, may be the principal concern of dental health care professionals and researchers, however it probably is not a major concern for people in everyday life. Hence, instead of focusing on impractical and infeasible (health) endpoints that are not germane to the periodontal fitness of people, a more rational approach is proposed. It defines periodontal health using a modified wellness model. Its principal paradigm would consist of four cardinal characteristics including: 1) a functional dentition, 2) the painless function of the dentition, 3) the stability of the periodontal attachment apparatus and, 4) the psychological and social well-being of the individual. Moreover, a periodontal apparatus that is not deteriorating in an individual who is satisfied with their function and appearance suggests a balance between mind, mouth and spirit. It reflects periodontal health.

This definition of periodontal health is a hybrid between the natural and the holistic concepts of health that reflects a notion of wellness. From the natural concept of health, the absence of progressive attachment loss around the tooth is a required objective characteristic. Effortless occlusal function reflects the holistic concept of health in that function and comfort do not have to be ideal but are relative to patient needs and wishes. Finally, social and psychological wellbeing (i.e., how I look, how I speak, how I feel, etc.) revolves around a personal constructive attitude, signaling a positive quality of life for periodontal wellness.

Notably absent from the proposed health definition are gingival inflammation and probing depth. Gingival inflammation is a well-known feature that is commonly found in the periodontium [11]. In fact, the prevalence of gingivitis in the adult U.S. population was estimated to range between 50% (CDCP) and 94% [12], depending on the clinical definition of gingivitis and the sample population that was studied. The pervasiveness of this condition would make one consider it to be endemic. It could be easily argued that the natural occurrence of gingival inflammation describes a condition whose characteristics are normally distributed in the population. Although gingival inflammation has been hyped as a gateway to serious periodontal conditions [13,14] and tooth loss [15] most gingival inflammation does not convert to destructive forms of periodontal disease and, conversely, some investigators have demonstrated loss of the attachment apparatus that appears to be independent of visible inflammation [16]. These factors call into question the clinical significance of plaque-induced gingivitis as a disease [13,17] and the putative role gingival inflammation plays in other forms of periodontal disease.

The second criterion not considered in the definition of periodontal health is critical probing depth. Although periodontists and periodontal investigators have used critical probing depth as case definition for destructive periodontal diseases [18,19], the probing depths may have nothing or everything to do with the historical occurrence of periodontal disease. To be accurate, destructive periodontal diseases, such as chronic or aggressive periodontitis, can only be truly diagnosed when active (i.e., in the presence of notable attachment loss over time). Accurately predicting or identifying individuals who will exhibit attachment loss is currently not possible with contemporary scientific or clinical methods. Therefore, someone with significant probing depths may have invariable attachment levels for months, years or a lifetime [20]. Hence, a reduced periodontium and/or a critical probing depth is primarily a sign of disease history and does not indicate the presence of active disease nor does it predict future attachment loss accurately and reliably.

## Periodontal health model

The proposed model for periodontal health is not concerned with disease processes as much as it is a paradigm for maintaining periodontal health. It outlines what factors affect the health of the periodontium (i.e., function, comfort, stability and well-being). Comfort, function, and positive sense of welfare are defined and expressed by the patient; however, stability of the periodontium requires observation by the dentist. In essence, a stable periodontium is one in which the attachment level is not changing as measured clinically with a periodontal probe. Although there are numerous limitations of using clinical attachment levels to determine periodontal stability, currently this is the gold standard used to determine change in the periodontal attachment apparatus [21].

**Figure 2:**
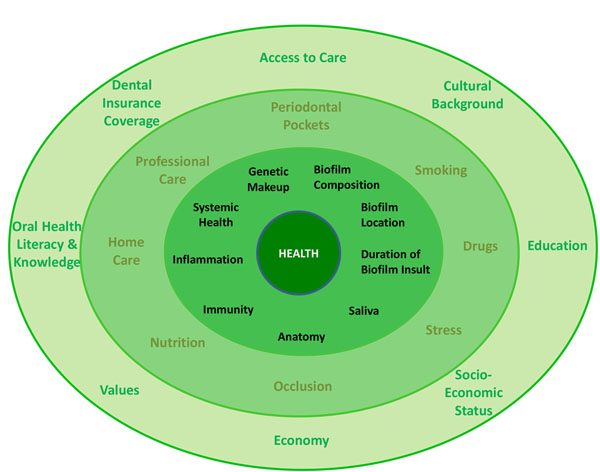
Three-Layer Periodontal Health Model (inner layer: directly contributing factors; middle layer: oral environment & systemic factors; outer layer: personal factors).

To tie the four cardinal signs of periodontal health together, a model (Figure 2) was constructed that contains three layers of factors that can influence periodontal health. The layers are: a) biologically discrete entities that have a direct effect on the periodontium, b) environmental and systemic factors that can influence biological components, and c) general modifying conditions (i.e., personal characteristics), which can influence both the biological and environmental/systemic factors. Obviously, by exclusively controlling biological factors, periodontal stability can be obtained; however, in the complex societies in which we live, the interaction of cultural, social, political, economic and personal factors often create situations where periodontal wellness is difficult to achieve without considering how to manipulate the general modifying factors. The factors itemized in the three layers have extensive data in medicine and dentistry to support their consideration as features that can substantially influence periodontal health. The salient evidence for each of the factors in the periodontal health model is presented.

## First Layer - Biologically discrete entities of biofilm and host that affect the periodontium

### Composition and character of the oral biofilm

Microbial biofilms cover skin, gut, uro-genital tract, naso-pharynx, and mouth of human beings. The bacterial composition of these biofilms is habitat-characteristic, diverse, and stable for core species [22]. Together with the surroundings of their habitats, biofilms form dynamic ecosystems. They are essential contributors to tissue homeostasis and health, but can contribute to or cause pathology when they are under significant distress. Maintaining balanced ecosystems has been proposed as a strategy to prevent disease [23].

Molecular techniques like polymerase chain reaction and pyrosequencing-based profiling have enabled the isolation of bacterial 16S rRNA gene sequences and classification of bacteria from biosamples without using traditional culture techniques [24,25]. Using these tools, more than 1100 taxa were identified from oral mucosa, tongue, saliva, tooth surfaces, and periodontal pockets. The results have been compiled in the NIH-funded “Human Oral Microbiome Database” (HOMD). The HOMD has enabled the definition of taxonomic cores within and across oral habitats and individuals [26].

Colonization of dental surfaces by bacteria is initiated by gram-positive aerobic commensals (e.g., *Streptococci, Actinomyces*) followed by more fastidious gram-negative bacteria (e.g., *Fusobacteria, Prevotellae, Treponemae*), ultimately leading to complex, sticky biofilms ("dental plaque"). Bacteria in biofilms can communicate with one another using cell-cell signaling systems and form multispecies communities that allow horizontal gene transfer. As a result, biofilms can resist mechanical and biological insults more easily than planktonic bacterial cells [27,28]. Suspected pathogens (e.g., *Treponema denticola, Porphyromonas gingivalis, Tannerella forsythia* [29]) are usually absent or found in small numbers [30].

Inflammation of the gingiva, i.e., gingivitis, is considered to be the result of a non-specific proliferation of the indigenous microflora residing in the gingival crevice [13]. Given currently poorly defined circumstances, the previously mentioned and many other pathogens (e.g., *Treponema socranskii, Filifactor alocis, Dialister pneumosintes, Porphyromonas gingivalis*) can emerge and form bacterial consortia with tissue-destructive capability [31]. Although most biofilm species have not been fully characterized, typical oral pathogens can affect the host response, have stealth capability, produce toxins, or invade eukaryotic cells where they can modify gene expression. Such properties are characteristic for agents that cause inflammation and tissue destruction [32].

### Location of biofilm

Dental biofilms develop preferably in areas where they are protected from the disruptive shear forces of flowing saliva, mastication, or cleansing. Preferred sites for initial colonization include coronal fissures and pits, enamel scratches and cracks, overhanging restorations, and gingival sulci (i.e., the narrow grooves between the gingival margin and the tooth surface). In the interproximal space between teeth, the gingival sulcus takes on the shape of a col that is covered by a thin, non-keratinized epithelium. In the presence of bacterial biofilms and inflammation, the (junctional/col) epithelium will proliferate and ulcerate, and the gingival sulcus may deepen to form a gingival pocket or, given special circumstances, a periodontal pocket, the cardinal sign of periodontal diseases [11].

Cleaning interproximal spaces and proximal tooth surfaces is a challenge for most people. As a result, biofilms can develop more easily on proximal surfaces near the col, possibly leading to tissue injury and, sometimes, to periodontal breakdown. Periodontal health can be restored and maintained by the assistance of dental professionals who are equipped for removing plaque-retentive factors and potentially harmful biofilms from difficult to access areas, for example in deep pockets [33].

### Duration of biofilm insult

Periodontal destruction was believed to start at a young age as gingival inflammation, ultimately leading to tooth loss if untreated as the person is aged. Older studies [34,35] suggested a direct, proportional relationship between plaque amount, level of inflammation, and tissue destruction. More recent longitudinal studies, however, revealed a subtler story [36]. In a cohort of adults with no access to professional dental care and minimum personal oral hygiene, it was shown that only few individuals experienced rapidly progressive destruction leading to tooth loss. Other participants were barely affected or even showed no destruction despite the presence of copious dental plaque deposits and inflammation. The study showed that periodontal breakdown was not an inevitable consequence of biofilm-induced chronic inflammation, and periodontal long-term stability could be observed in the absence of perfect oral hygiene.

The presence of biofilms in periodontal pockets with ensuing inflammation has been suggested to predict future periodontal destruction. However, clinical studies showed that bleeding on probing - a reliable indicator of inflammation - was a poor predictor for future periodontal destruction. Only sites that were inflamed over a very long time span had an increased risk of breaking down [37,38]. In contrast, and not unexpectedly, sustained absence of inflammation was a reliable predictor of periodontal stability [37,38].

The health of a living system involves properly functioning homeostatic mechanisms and the appropriate response to the presence of foreign materials. In the oral cavity, numerous physiologic activities are present that support health and protect the individual from illness and disease. The oral cavity is unique considering that it is the only area of the body where calcified objects are projecting out of mucosa into an environment bathed in fluid, growth factors, enzymes and bacteria. Moreover, health of the host ultimately depends on the genetic makeup of the individual and how various environmental factors affect gene expression. In the following, the role of the “host” factors is described.

### Saliva

Secretions produced by major and minor salivary glands and gingival crevicular fluid constantly bathe teeth and oral mucosa. They keep oral tissues wet and healthy. The functions of the saliva are varied and complex. Regarding periodontal health, saliva provides antimicrobial potency via specific (e.g., IgA, etc.) and non-specific (e.g., lysozyme, lactoferrin, etc.) mechanisms, regulation of plaque pH, agglutination of bacteria (e.g., mucoglycoproteins), growth-modulating effects on bacteria, and elimination of bacteria and nutrients from the oral cavity. Since saliva can modify the supragingival biofilm [39], subgingival microflora [40] ultimately can be affected by saliva composition, too. Although reduced salivary levels (i.e., hyposalivation) may be associated with periodontal disease [41], periodontal disease develops in individuals regardless of their salivary flow and composition; therefore, saliva composition alone does not prevent periodontal disease. However, even mild forms of hyposalivation can result in mucosal pathology that affected patients sometimes described as a “burning sensation of the gums". Hence, proper salivation is a contributor to periodontal wellness.

### Anatomical characteristics of alveolar bone and teeth

There are conditions occurring to both hard and soft tissues in the oral cavity that may affect the stability of the periodontium and potentially increase the risk of disease. The tooth-related factors that can contribute to the accumulation of bacteria and affect periodontal health may include cervical enamel projections, enamel pearls, tooth position, root proximity, open tooth contacts, root abnormalities, furcation anatomy and location, restorative marginal discrepancies, restorative materials, and tooth fractures [42]. In addition, the thickness of the alveolar bone on the buccal and lingual tooth surfaces has been implicated in the etiology of gingival recession and disease progression [43].

### Immunity

Maintaining periodontal health involves a complex immune response to the tooth biofilm. Since surveillance by the immune system protects an individual by recognizing and responding to antigens, when the immune system is overwhelmed, clinical symptoms to disease become evident. The periodontal health and conversely the susceptibility of a person to periodontal disease result from an inflammatory response as well as the activation of various immune pathways that involve the regulation of both innate and acquired immunity [44]. Therefore, the responsiveness of the immune system in combination to the amount and type of various antigens can affect the health of the periodontium. Individuals with defective immune responses (e.g., localized aggressive periodontitis, etc.) as well as those with hyper reactive immune responses can have their periodontal health affected [45].

### Inflammation

Although gingival inflammation has been proposed to be a critical factor for progression to more destructive forms of periodontal disease [46], not all forms of inflammation appear to be deleterious. Acute periodontal inflammation is important for the protection of or healing of tissues. Even when considering chronic forms of gingival inflammation, not all gingival inflammation progresses to periodontitis [47]. There are further paradoxical reports of a form of periodontitis that develops independently of clinical evidence of inflammation [16]. Further, the ability of a clinician to use the inflammatory status of an individual to predict future loss of attachment around teeth is poor [37]. Therefore, the sole presence of inflammation does not necessarily mean disease but rather, a physiologic adaptation of healthy tissues to biological insults that are regulated by the genome. To be sure, the chronic inflammatory mechanisms resulting from the relationship of environment (e.g., diet, stress, bacteria) and genetics (e.g., specific mechanisms to restore homeostasis, gene expression in tissues, inflammatory response) creates a complex dynamic that does not include nor exclude inflammation as an arbiter of health or disease.

### Systemic health

A multifaceted relationship exists between the systemic and periodontal health of an individual. There are systemic factors, involving inflammatory and immune reactions that can disrupt the periodontal health of an individual. The putative role of systemic conditions in challenging or modifying the health of the periodontium has been associated with a decline in gingival health and overall health of both hard and soft periodontal tissues. More specifically, endocrinopathies (e.g., diabetes mellitus), immunosuppression (e.g., acquired immunodeficiency syndrome), hematologic disorders (e.g., neutropenia) and genetic disorders (e.g., Down's Syndrome, leukocyte adhesion deficiency syndrome) have been linked to the destruction of the periodontium [48]. Although systemic health does not directly confer health to the periodontium, there are some systemic disorders, in particular diabetes mellitus, which can directly affect periodontal health and wellness.

### Genetics

Although it has been posited that genetic factors play an important role in periodontal health and disease [49,50], the relationship between the genetic composition of the individual and the health of the periodontium is complex. In regard to disease, genetic contributions can be broadly categorized into two types of action. In one instance, the genetic influence on etiology can be the driving force that determines the phenotype of the disease. When this occurs, a significant alteration in gene function (i.e., a mutation) results in a protein or proteins that characterize the disease phenotype. Such mutations are transmitted in accordance with Mendel's laws of inheritance. Unlike Mendelian diseases, the other category of gene action involves complex or multifactorial genetic influences in the population. More specifically, complex or multifactorial genetic conditions arise as a compilation of the actions of multiple genes, each contributing to a relatively small portion of the etiology. These multiple genetic contributions must act jointly with appropriate environmental factors (e.g., microbes, smoking) to trigger disease when a particular ceiling of genetic and environmental elements is present. In regard to periodontal disease, multifactorial genetic influences are more prevalent in its etiology [51]. What becomes even more complicated than the affiliation between genes, environment, and disease is what genetic influences are necessary within the environment to promote periodontal health. Furthermore, epigenetic modifications by the environment have been mentioned to also affect the expression of periodontal health [52,53]. Obviously, numerous genetic characteristics for the oral cavity (e.g., type and quality of bone surrounding the tooth root) and epigenetic modifications are important factors that determine host response to possible insults and confer susceptibility to periodontal health.

## Second Layer - Environmental and systemic factors that can influence biological entities

### Pre-existing periodontal pockets

The presence of residual probing depths greater than 4 mm has been implicated as a risk of future attachment loss. Although high frequencies of deep residual pockets and/or deepening of periodontal pockets have been associated with additional attachment loss [54,55], longitudinal studies have established that deeper probing depths may remain stable depending on the individual maintenance care of the patient [56,57]. Similar to gingival inflammation, probing depths alone should not be considered detrimental to periodontal health unless placed into context with other clinical parameters such as, repeatedly occurring bleeding on probing, and anatomical conditions that allow for ecological niches of bacterial infection [58].

### Smoking

There are many studies that implicate cigarette smoking as one of the significant modifiable risk factors for periodontal destruction [59-61]. More specifically, it has been reported that heavy smokers are facing a two- to eight-fold increased risk for periodontal attachment loss and/or bone loss as compared to non-smokers [62]. Further, the amount of lost bone was dependent on the number of cigarettes smoked [62]. It is not surprising that smoking cessation diminishes the risk of initiation and progression of destructive periodontal diseases and improves the response to periodontal therapy [63]. Abstinence from smoking is an important factor in maintaining periodontal health.

### Drugs

The most common changes to exclusively affect the periodontium result in the enlargement of gingival tissues as a consequence of using an anticonvulsant (e.g., phenytoin), an immunosuppressant (e.g., cyclosporine A), specific calcium channel blockers (e.g., nifedipine, verapamil, diltiazem, sodium valproate) or sex steroid hormones (e.g., oral contraceptive agents) [13,64]. In some instances, the overgrowth of the gingival tissues can be esthetically disfiguring and can affect chewing function. The etiology of these drug-influenced changes is not well understood but usually involves anterior gingival segments that are first observed in the gingival papilla [13]. These lesions are not directly associated with attachment loss or tooth mortality [13].

Vesiculobullous disorders, such as erythema multiforme, Steven Johnson syndrome, anaphylactic stomatitis, lichenoid drug reactions, and pemphigoid-like drug reactions, can also be the result of an untoward reaction to systemic drug administration. The different conditions can have different clinical presentations that are not confined to the periodontium and may involve skin as well as induce widespread mucous membrane lesions. Although the oral lesions can be painful and affect oral function, they generally have no effect on the periodontal attachment apparatus. However, they may affect oral wellness.

Reduced salivary flow is a frequent sequel of autoimmune diseases (e.g., Sjögren's Syndrome), -irradiation (e.g., head and neck carcinomas), medications (more than 400 drugs that have been recognized), and developmental disorders (e.g., ectodermal dysplasia) [65]. Hyposalivation has been implicated in increasing the risk of root caries, dental erosion, dental hypersensitivity, chronic mucositis, oral candidiasis as well as destructive periodontal diseases [41,66]. In addition, similar concerns apply as listed in the “Saliva” section.

### Stress

Psychological stress has been implicated as a contributing factor in a variety of diseases, including depression, cardiovascular disease, and asthma, to name a few. It is known that chronic stress can affect the immune system, wound healing [67] and contribute to pathogenic infection potentially leading to destruction of the periodontium in susceptible patients [68]. Unfortunately, the complex biological nature of stress complicates our understanding of how it modulates periodontal health in conjunction with numerous environmental factors (e.g., biofilm, hygiene, diet, smoking). At this time, even though there is an incomplete understanding of how stress can modify the susceptibility or progression of periodontitis [69-71], how we cope with stress may play an important role in our periodontal well-being [72,73].

### Occlusion

The relationship of occlusion to periodontal stability continues to be a conundrum resulting from numerous and equivocal studies. Tooth mobility can result from a variety of factors with the mobility ultimately being a physiologic adaptation to the clinical forces [74]. Therefore, tooth mobility independent of periodontal inflammation is associated with periodontal health. In contrast, occlusal forces that produce progressive mobility in the presence of a biofilm-induced inflammation may modulate progressing periodontal disease [74,75]. However, most laypeople associate severe tooth mobility, irrespective of etiology, with periodontal pathology. They may experience gingival pain even when applying small bite forces and their chewing ability can be seriously impaired. In the affected person's mind, untoward tooth mobility is a serious concern that may shift the scale from periodontal wellness to illness.

### Nutrition

It has been postulated that a compromised host defense system resulting from malnourishment can significantly exacerbate the response of the periodontal tissues to biofilm bacteria [13]. Unfortunately the precise role of nutrition in the initiation or progression of periodontal diseases remains to be elucidated in humans [13,76]. Perhaps of all the nutritional deficiencies observed in the oral cavity, the clinical description of severe vitamin C deficiency or scurvy has been one of the earliest and best documented [13,77]. Therefore, adequate provision of vitamin C appears to be a prerequisite for periodontal health. Similar to vitamin C, antioxidants are another possible nutritional component that may be associated with periodontal health. A recent evaluation of NIHANES III data demonstrated a relationship between serum levels of antioxidants and periodontal health [78]. Although a proper diet is important for long-term human survival, the existing scientific evidence to recommend specific micronutrients for oral health is insufficient [76].

### Home care

The importance of personal oral hygiene to remove supragingival plaque has been the central paradigm of periodontal care. Considering that the major modifiable risk factor for periodontal diseases is the dental bacterial biofilm, the apt removal by the patient on a regular basis becomes paramount [44]. Consequently, there has been considerable evidence that mechanical removal (e.g., tooth brushing, flossing) of the dental biofilm can dependably affect stability of the periodontal tissues [33,79,80]. Properly performed oral hygiene requires highly motivated, skilled individuals with sufficient dexterity, effective cleaning devices, chemotherapeutic agents for plaque removal, and appropriate oral hygiene instruction.

### Professional Care

Very few individuals can maintain periodontal health and prevent tooth loss without the benefit of regular dental care by a dental health care provider [81,82]. Intervention by a health care provider, delivering either nonsurgical and/or surgical periodontal therapy to individuals with active periodontal diseases has been shown to improve long-term retention of teeth [44].

Following periodontal therapeutic interventions, patients who fail to have maintenance care at defined and regular intervals have more tooth loss than those patients who receive defined supportive periodontal care [83,84]. Moreover, clinical investigations have shown that tooth loss was related to the frequency and quality of maintenance care in individuals with periodontal disease [85,86]. A systematic review confirmed that patients who were seen at regular intervals for supportive periodontal therapy experienced less attachment loss and lost fewer teeth [87]. Therefore, it is extremely important for a patient to retain periodontal health by receiving comprehensive periodontal maintenance treatment at appropriate intervals following active periodontal therapy [44].

## Third Layer - General conditions that influence both the biological and the environmental / systemic factors

### Access to care

Although periodontal diseases can affect people of all ages and backgrounds, millions of individuals are not able to obtain basic dental treatment. For instance, more than 47 million Americans live in places where dental care cannot be routinely accessed. Specifically, low-income, racial or ethnic minorities, geriatric adults, people with special needs, pregnant women and individuals from rural communities have fundamental struggles accessing a dental provider. While many reasons contribute to reduced access to care, some of the problems include a perceived lack of importance, dental phobia, difficulty searching for providers, limited available providers, struggles arranging appointments, and obstacles finding transportation [88]. When dental care has been accessible, there has been a noted positive effect on periodontal status of those individuals receiving attention [89]. In essence, people who use quality dental care services are more likely to enjoy better periodontal health than those who don't have access.

### Cultural background

The shared actions and beliefs that provide social contexts to define behavioral norms for relationships and a proper lifestyle in a society often are referred to as culture. It would be anticipated that the traditions and customs of society play an important role in people's health. In other words, cultural differences influence the response to disease symptoms, pursuit of medical care, and perception of the health care team [90]. As a result, all this affects how an individual will react and respond to an ailment and its treatment. For example, Chinese health beliefs that emphasize traditional Chinese medicine suggest that an affected meridian (energy pathway) results in periodontal disease [91]. The use of traditional Chinese medicine to cure the disease often leads to a strong reliance on self-care and delays seeking care by western biomedical dental standards [91].

In regard to cultural background, health disparities between gender, race and/or ethnicity have been correlated with persistent differences in the incidence, prevalence, mortality and burden of disease. For example, native Americans have been 2.6 times more likely to be diagnosed with diabetes when compared to non-Hispanic whites [92]. In regard to periodontal diseases, higher rates of aggressive periodontal disease have been reported in African-Americans when compared to other racial groups [93]. Consequently, the struggle to eliminate health disparities has involved understanding how disease distribution relates to socio-political structure, discrimination, cultural practices, socioeconomic status, exposure to toxins, and access to health care [94-96].

### Education

The association between the amount of education and health status has been well-documented for a broad assortment of health measures [92]. There is an inverse relationship between mortality and years of schooling as well as poor health status and amount of education [97]. The association between education and health is a complicated relationship that cannot be fully explained by income, the labor market, or family background indicators [92]. As a result, there is an expansive range of potential mechanisms influencing the relationship between education and health. Similar to systemic health, the periodontal health of an individual also exhibits a positive relationship with the years of schooling [98,99].

### Socio-economic status

The economic and sociological measure of work experience, social position and economic influence formed by an individual's income, education and occupation has been recognized as the socioeconomic status of an individual or family. Inequalities in socioeconomic status have been considered a fundamental issue affecting health. It has been shown that the higher the socioeconomic status of a person, the better health, less disability, less disease and longer life they will live [100]. Similar to medicine, there are socioeconomic gradients in oral health. Inequalities have been demonstrated in tooth retention [101], decay [102], and restoration [103] as well as periodontal disease [104-110]. The causal pathway by which socioeconomic status improves systemic or periodontal health has not been well established, considering that socioeconomic gradients in health are multifactorial and inter-related.

### Economy

The economic status of a locale will govern the extent to which resources can be generated for a healthcare system as well as the ability of the residents to use the system. Furthermore, the economic status will dictate policy options to structure and organize the healthcare system. It is interesting to note that high-income areas can afford to base their healthcare system more on specialty care than on primary care [111] and public satisfaction is often higher in regions offering directly accessible specialist care [112]. In regard to the individual, the state of the economy has a direct impact on employment that affects how people prioritize their health care needs and how they disperse their health care capital.

### Values (attitudes and beliefs)

What society values as essential for health may explain differences in healthcare policy priorities, services delivery, healthcare utilization and outcomes [113-117]. In the first place, the political values of a state will affect how health care is provided. Regions that have a socialist government aim to achieve equity in a redistributive social system with generous benefits. The opposite is true for conservative governments that aim to achieve equity in a merit-based social system with benefits being compensated by the individual and not the state. Previous research has shown that a region's political configuration is correlated to healthcare system policy priorities [118-120]. Secondly, the value systems of health care professionals towards patients will determine the health status of the individual. Overall, a provider may achieve high value health care for a patient that revolves around patient needs in an economically sustainable health care system [121]. The third piece of the value system and perhaps the most important one is the attitudes, beliefs and preferences of the patient. Moreover, health care values of the patient provide direction in choosing treatment options and designing interventions specific for the individual [122].

### Oral health literacy and knowledge

**Figure 3:**
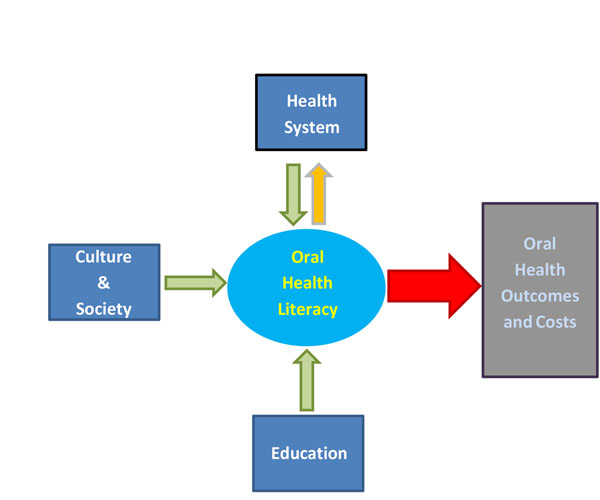
Oral health literacy framework (Adapted from IOM, 2004 [125]).

Oral health literacy was defined in Healthy People 2000 as “The degree to which individuals have the capacity to obtain, process and understand basic health information and services needed to make appropriate oral health decisions” [123]. Therefore, the ability to acquire, read, comprehend and appropriately use oral health care information to value judgments concerning health care options requires health literacy. In addition to education, oral health literacy is impacted by the complexities of the health system, cultural influences, and societal values (Figure 3).

Although health care information is necessary for decision-making, the Institute of Medicine [124] described a significant discrepancy between information people receive and what they understand [125]. Accordingly, 30% of U.S. parents have difficulties understanding and utilizing health information [126]. The incongruity between what is said and what is heard results from limitations of the person receiving the information and the health care provider delivering the information. For the health care provider presenting the information, health information becomes difficult for an individual to understand because of the complexity of the information presented, unfamiliar jargon used, and the demands of navigating the health care system (e.g., finding providers, bureaucracy, etc.). Although even highly educated individuals struggle to comprehend health care information fully, understanding the concepts regarding disease initiation and progression, prevention and treatment can be challenging for people with limited capacity to obtain, process and understand basic information and services needed to make suitable decisions for their health [124,127]. By understanding these caveats, it was not surprising that periodontal health was found to be associated with individuals who had a high oral health literacy score [128].

### Dental insurance coverage

Approximately 40 percent of working age adult Americans visit the dentist on a yearly basis [129] and although the reasons for utilization of dental services are complex, one of the principal excuses for limited utilization rates is the expense of dental care [130]. It has been estimated that 44.2 percent of dental costs are paid directly by the consumer, whereas only 10.3 percent of physician costs, 3.3 percent of hospital care cost, and 26.8 percent of nursing-care expenses were paid out-of-pocket [131-133]. Further, the projected average annual out-of-pocket costs for dental care were over $800 [130]. Consequently, the cost of dental care is often reported as a barrier to dental treatment. In the United States, the dental insurance provided either by an employer, a private insurer or the federal government reduces but does not eliminate the barrier of dental utilization. For those with dental insurance, 49 percent were more likely to visit the dentist and 42 percent were more likely to take their children to see the dentist [134]. Finally, besides increased utilization, those with dental insurance had more predictable dental expenses and were less subject to high dental costs [130]. Consequentially, by having dental insurance, the access to dental services can substantially improve health outcomes.

## How periodontal health can be promoted

There is an urgent need for strategies and policies that advocate periodontal health. These policies and strategies should be guided by an understanding of how much activity and through what specific actions dentists can take to reduce periodontal disease risks through the view of the routine nature of daily life, with all of its own pleasures, pressures, constraints and potential rewards and risks. To affect the periodontal health of the individual and society, several strategies can be considered.

## Changing behavior to promote prevention

Both public dental health and dental health care systems at various stages of development face the need to prioritize policies and programs as they relate to prevention and treatment, financial and workforce issues, as well as in ways to improve people's periodontal health. Improving and supporting effective periodontal preventive measures require more than just an extensive understanding of prevention and detection of disease risk for an individual; rather, it requires substantial understanding of the modification of behavior in the general population.

It has been suggested that human behavior is not propelled by a calculation of consequences of actions but is the consequence of actions independent of conscious reflection [135]. Patients may act in a reflective manner, (e.g., aiming towards goals, modifying behavior to reach goals) or in an automatic or habitual manner (e.g., acting without reflection, responding without thinking about the surroundings), and these two behavioral actions often overlap and interact creating a complex mixture of the two behaviors. This is particularly true of dental behaviors in which people have competing goals. For example, the pleasure of eating candy versus the dental health goal of reducing sugar intake and intake frequency to prevent caries. Despite the dominance of habits in directing behavior, the interventions for preventive periodontal health services primarily target the reflective process. Current preventive periodontal services range from informational interventions (e.g., pamphlets, brochures, lectures) to imparting complex skills to engage in increased self-regulatory behaviors (e.g., tooth brushing/flossing methods, visits to dental health care providers). Unfortunately, the potential to change behavior in a large enough sample of society is limited since only a small percentage of people with unhealthy behavior engage in preventive programs and those that do engage are usually already healthy resulting in modest positive effects [136].

Altering environments to constrain the influence of negative habitual processes must be considered for periodontal health. For example, improving the effort to remove tooth biofilm by reducing the necessary exertion, developing more pharmacologically potent toothpastes, increasing healthy food/drink choices by vendors, and designing oral hygiene products to positively shape behavior are some things that can be done immediately. Affecting habitual or automatic associative processes will be more difficult to target in society. For example, changing plaque removal practices that are limited and inadequate (e.g., not brushing or brushing for limited periods of time) **will require positive and consistent encouragement to assure the goal will be achieved permanently**. Another method to change habitual processes involves altering or creating new positive associations for healthy behaviors. Using fun terms to describe healthier foods, preventive behaviors, or interactions with health care providers increases the chances of individuals using preventive periodontal care. An illustrative example is the “tooth friendly” or “safe for teeth” label of non-cariogenic food items that is very popular in Europe [137]. To reduce habitual health damaging behaviors, health care providers will need to overcome significant philosophical, regulatory, political, and economic barriers if periodontists are to gain a foothold in the multitude of existing cues that drive health-harming behaviors.

## Improve oral health care literacy

Save, effective and successful use of services, self-care, and maintenance of periodontal health is dependent on oral health literacy. Furthermore, if individuals are to have an active role concerning the decisions and management of their health, oral health literacy becomes a fundamental requirement. Unfortunately, a significant portion of the adult population lack health literacy skills to understand and act on information related to their health [138,139]. Since oral health literacy is fundamental to improving the quality of health while reducing expenses and eliminating disparities among individuals, several simple steps could be considered that can be easily instituted by third party insurance carriers and/or government. These steps include incentivizing dental homes (i.e., private and public dental care facilities) to be health literate, supporting the public access to essential health documents, expanding oral health literacy skills and competency in the workforce, utilizing technology to shift communication platforms that are used extensively and encouraging efforts in prevention, including home care, as well as health care coordination that facilitate an oral health literacy approach.

## Engineer more efficient, safe chemotherapeutic agents

The periodontal microbiota has been considered as an infection in the gingival tissues resulting in an inflammatory response with possible destruction of the attachment apparatus. Rinses with chemicals, for example chlorhexidine, can substantially reduce dental biofilms when used regularly and at an appropriate concentration [140]. However, adverse effects, like staining and alteration of taste sensation, and poorly understood or unknown consequences associated with the chemical's long-term use have led to the virtual disappearance of the commercial product from the shelves of retailers. Instead, other, less potent ingredients, which were subject to less scrutiny by federal regulatory or private agencies, were incorporated into mouthwashes and toothpastes and are still available. It is interesting to note that when a particular drug is introduced to the oral cavity to affect the tooth biofilm or amount of inflammation, dentists become “giddy” when there is a 20% reduction in plaque or inflammation. The same could not be said if there was a similar reduction in a *Mycobacterium tuberculosis* lung infection. Clearly, chemicals that do not affect the delicate ecological balance of the healthy oral microbiome continue to be the greatest hope for the development of a potent biofilm-reducing agent. However, the development of a product for home use, which combines high potency in the presence of absolute safety and at low cost, as demanded by modern society, will be an enormous challenge. This challenge is further complicated by government bureaucracy and the expenses necessary to bring such a product to the masses. As part of the greater good for society, public funds combined with relaxation of the bureaucracy, in connection with private industry, must be brought to bear to create a safe and effective agent for maintaining periodontal health.

## Innovate plaque removal technology

Removing dental biofilms and tooth stain completely and without tissue damage is the obvious yet hard-to-reach objective of home care tooth brushing. Manual and powered toothbrushes, in combination with sophisticated dentifrices, provide substantial support towards achieving the highly desirable result. Residual biofilm on proximal surfaces can be removed with dental floss or mini brushes, but the successful execution of the task is challenging most people for its exceedingly high level of manual skill and dexterity. In addition, dental floss does not reach into the anatomical concavities of proximal surfaces of premolars and molars and thus renders the effort incomplete [141,142]. Water and air supported powered flossing devices represent progress in the right direction, but they are somewhat messy in their use, too expensive to buy for most people, and still do not remove biofilm completely. Of course, it is currently not known whether the complete removal of biofilm daily is even necessary to maintain periodontal health long-term or whether partial biofilm reduction, as achieved with regular, traditional oral hygiene, would be as effective. Further, complete biofilm removal appears to be essential for achieving esthetically pleasing, shiny teeth, which for most people will always be the ultimate reason and motivation to spend time and money on oral hygiene measures. Hence, there is a need for innovating oral hygiene through the development of technologies that go beyond the simple mechanical cleansing effect of bristle tips. More specifically, the use of novel technologies to sense the oral environment and navigate around teeth without human input to deliver safe, effective, and inexpensive plaque removal would deliver oral cleanliness as part of a holistic wellness program.

## Ascertain patient risk factors

Knowing and understanding a patient's risk level for periodontal disease would be a key information piece in the management of their periodontal health. Many potential risk predictors have been identified but, with few exceptions, they have shown poor predictive power. History of smoking, history of untreated periodontal disease, and poorly controlled diabetes are examples of well-documented predictors of future periodontal destruction [143]. Risk prediction instruments that combine the might of several potential risk factors and indicators have been presented [144,145]. They are available to the dental profession for practice use. In the future, the value of such tools could be enhanced and personalized by the incorporation of biochemical content, pertinent information obtained from the patient's genetic screen, electronic patient record, and oral microbiome composition.

## Strategies for early detection, useful diagnostics, and non-invasive treatment

Depending on the patient's risk profile, more or less frequent visits to the dental office are essential for the patient management. Similarly, the dentist's ability to detect early signs of instability of the periodontal attachment apparatus with high diagnostic accuracy, will allow for appropriate care for the maintenance of a patient's periodontal health. The periodontal probes used in practice today are inapt to execute the proposed task. Probing should be replaced with quantitative imaging techniques that impress with excellent sensitivity and specificity, and are fully compatible with future electronic patient records. Efforts to treat periodontal disease should focus on the four elements of the periodontal health model with the goal to manage the disease process and re-establish health, always considering the patient's needs and wishes while avoiding over treatment.

## Industry - community engagement

Industry's contribution to the development of dentistry as a scientific discipline and profession might be obvious to only a few people, but there is good evidence it has been enormous. Collaborations between industry and academia are common in dentistry and have the potential to produce excellent research. Based on consumer and professional feedback, many product ideas of high commercial value were developed and introduced. For example, besides selling toothbrushes, toothpastes and mouthwashes, industry engaged in the development and marketing of items that are helping improve diagnosis (imaging, probing), pain control (local anesthetics, sedation), tooth substance and tissue management (high-speed drill, laser), esthetics (tooth whitening, orthodontic appliances, restorative materials), function (orthodontic appliances, implants), and practice administration (computing, networking, electronic patient record). However, possibly more than at any time before, change in dental practice is motivated by advancements in technology primarily, rather than better understanding of human biology and physiology.

Similar to the private sector, government has historically influenced health care policy as well as health care costs. Issues relating to dentistry that have been influenced by government agencies have involved professional standards, health planning, public health, financing, prevention, research, and manpower to name just a few. Although periodontal health is substantially affected in the population and would benefit from increased support, the funds provided by government sources is limited when considering that other, more debilitating chronic diseases are also in need of financial support, and combined with pressures for economic support of education, labor, defense, safety, social programs, etc., the priority for maintaining periodontal health becomes limited. Nonetheless, government can affect periodontal health by prioritizing periodontal projects at federal research agencies and providing tax credits to industrial partners.

## Implementation of Periodontal Health in Practice

There are two broad strategies for the implementation of procedural guidelines for health: one considering what can be done immediately and the other reflecting on future opportunities. Since activities and policies that can be instituted have been described in the preceding paragraphs, the following suggestions highlight what can be instituted immediately to contribute to periodontal health as well as a vision for possible, future developments that can substantially promote periodontal health (Table 1).

**Table 1:** Implementation of Periodontal Health in Practice

Periodontal Wellness Components	What Can Be Done Today	**Long-term vision(10+ years)**
**Periodontal Stability**	Periodontal diagnostics - attachment levels Risk assessment - questionnaire - health history Plaque control - professional care - home care Periodontal maintenance	Periodontal diagnostics - sensitive/specific - predictive Comprehensive risk assessment - biomarkers - systemic health - bone density profile - genetic markers Personalized comprehensive biofilm management Personalized periodontal maintenance
**Favorable Environment**	Lifestyle choices - nutrition - smoking cessation - stress management - systemic health Oral health literacy and knowledge Access to care - dental insurance	Lifestyle choices - nutrition - smoking cessation - stress management - systemic health Oral health literacy and knowledge - e-media (apps, etc.) - social media - science cafe Access to care - dental insurance - e-health record - comprehensive health management

In regard to what can be instituted immediately, preventive measures continue to play a major role in promoting health. Periodontal maintenance care has always been the cornerstone for maintaining functioning teeth in the dentition [146,147]. However, a variety of factors, including but not limited to cost, compliance, and lack of dental health care provider time contribute to shortfalls in the delivery of recommended treatment [148]. Therefore, personal home care and eliminating destructive behaviors (e.g., smoking) become important factors for periodontal health. It is well known that both short term improvement as well as long term improvement in plaque levels can be obtained in patients regardless of the mode of plaque control instruction but this must be individualized to the patient, otherwise, relapse will occur to baseline levels.

More specifically in regard to home care, the removal of supragingival dental plaque by effective tooth brushing plays an important role in the maintenance of periodontal health. Despite the importance of mechanical plaque removal as a safe and cost effective means to reduce supragingival plaque, patient compliance with frequency and duration of use has been lacking [149]. Therefore, powered toothbrushes with an integrated timer and other smart features are devices to promote periodontal health effectively. In addition to powered toothbrushes, the transport of active drug to the affected area in the oral cavity has had substantial and positive effects on periodontal health [142,150]. Considering that home care using mechanical plaque removal has limitations, the use of a drug to control supragingival plaque growth combined with reductions in oral inflammation can have positive effects on periodontal health [151-155].

Another factor that has an immediate effect on dental practice relates to the identification of and management of risk factors for disease. In general, a risk factor relates an aspect of personal behavior, lifestyle, a consistent environmental exposure, or an inherited trait with a disease or condition. In regard to what affects periodontal health, scientific evidence has demonstrated that smoking and diabetes mellitus can affect the stability of periodontal health leading to the initiation and progression of periodontal disease [156,157]. Furthermore, periodontal health is also at risk when access to care is limited, or patients do not have the socioeconomic means to pursue health care. Therefore, having patients aware of factors (e.g., smoking, diabetes mellitus) that affect their oral health as well as practitioners who are cognizant of elements (e.g., access to care, socioeconomic status, etc.) that place the periodontium at risk for disease will profoundly impact oral health outcomes.

One of the more significant aspects of care that immediately affects periodontal health is health literacy. Oral health literacy allows the patient to obtain services that are critical to their oral health management [158,159]. It is important to note that oral health literacy not only involves the patient but also the oral health provider as well as auxiliary personal. Areas commonly associated with oral health literacy include patient-physician communications, drug labeling (instructions and compliance), health information publications, informed consent, responding to medical and insurance forms, accurate recording of patient health history, and use of allied professional programs (e.g., social work, speech-language pathology, etc.). For the patient, improving reading skills, in addition to being involved in health literacy programs at libraries, schools or community groups can improve the ability to understand information and services for health.

Finally, reliably diagnosing ongoing attachment loss is an imperative and important activity for assessing periodontal health. Many clinical surrogates, including probing depths, attachment levels, alveolar bone levels and inflammatory status have been used to ascertain if periodontal destruction had occurred [160]. These clinical tests have limited prognostic power and are primarily indicators of periodontal disease history. A powerful advancement in the maintenance of periodontal health will involve tests that predict the decline of health leading to future periodontal destruction.

## Conclusion: a recipe for health

Defining health has never been a facile assignment. In fact, discussions concerning what constitutes health have evaded philosophers, politicians, educators, clinicians and many others for centuries. In modern dentistry, the approach taken has revolved around pathological conditions that can create death, morbidity or limits of activity. As a result, it is not surprising that dentistry, as well as medicine, have often been criticized for being, almost exclusively, disease oriented. Health, on the other hand, is something everyone wants to achieve. However, to be health oriented requires an understanding of what constitutes health. Like the proverbial elephant in the room, health can be “easy” to spot depending on your perspective yet establishing the concept of health has been a conundrum. As has been discussed, health is more than just the absence of disease; it is a personal, social, emotional and physical condition. The proposed definition of periodontal health revolves around a precept that a **stable** periodontium functions in comfort in an individual with psychological and social well-being about their mouth. Using such a simple definition for periodontal health, it is easy to focus on factors that put the health of the person at risk. In fact biological, environmental, systemic, social, economic and psychological factors can affect periodontal health in either positive or negative ways.

As individuals, health is what one wants to preserve for a life in comfort when combined with a sound body, mind and spirit. As health care professionals, patient health revolves around the modulation of behavior, preventive opportunities, speaking a common and easily understood jargon, and using pharmacologic, medical and dental technologies to preserve health. As educators and scientists, we hope these notions, concepts, thoughts, beliefs, facts, and philosophies will stimulate you to think and provoke a healthy debate about this subject.

## Authors’ contributions

AM and AFH drafted, read, and approved of the final manuscript.

## Competing Interests

AM received funding from Colgate Palmolive to attend the Prevention in Practice meeting in Capetown and has no other competing interests.

AFH: The author declares that they have no competing interests.

## Declarations

This article has been published as part of *BMC Health Services Research* Volume 15 Supplement 1, 2015: Proceedings of Prevention in practice - making it happen. The full contents of the supplement are available online at http://www.biomedcentral.com/bmchealthservres/supplements/15/S1. Publication charges for this supplement were funded by Colgate Palmolive.
